# Railroad Pattern of Atrial Intervals in a Dual-chamber Pacemaker Patient—What Is the Mechanism and How to Manage?

**DOI:** 10.19102/icrm.2022.130406

**Published:** 2022-04-15

**Authors:** Debabrata Bera, Anunay Gupta, Sanjeev Kathuria

**Affiliations:** ^1^Department of Cardiology, RTIICS, Kolkata, India; ^2^Department of Cardiology, Vardhman Mahavir Medical College, New Delhi, India; ^3^Department of Cardiology, GB Pant Hospital, New Delhi, India

**Keywords:** Far-field R-wave oversensing, railroad appearance, VA conduction

## Abstract

A 55-year-old woman with a dual-chamber pacemaker presented with brief episodes of rapid palpitation. The device recorded several stored atrial high-rate and ventricular high-rate episodes. The atrial intervals showed an interesting railroad track pattern during a non-sustained episode of ventricular tachycardia. We discussed the differential diagnosis of railroad track patterns on the atrial channel. In our case, it was related to far-field R-wave oversensing.

## Case presentation

**Figure 1:**
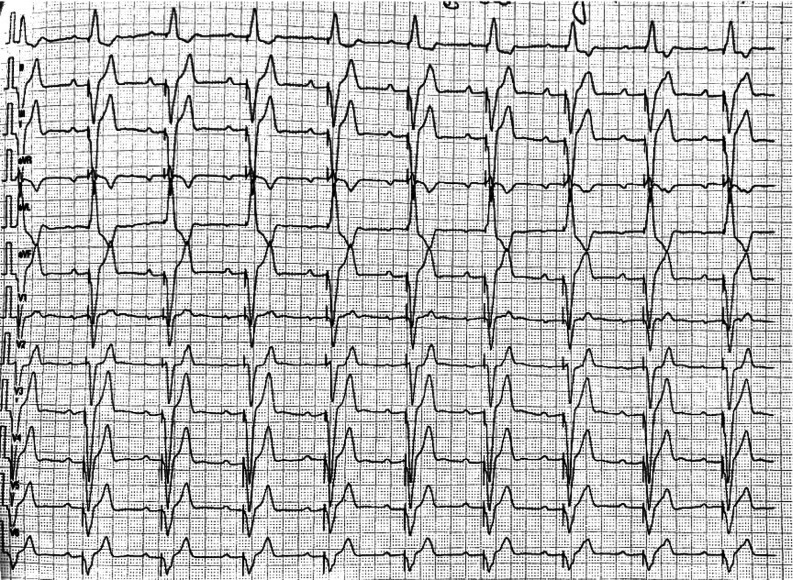
Surface electrocardiogram without magnet.

**Figure 2:**
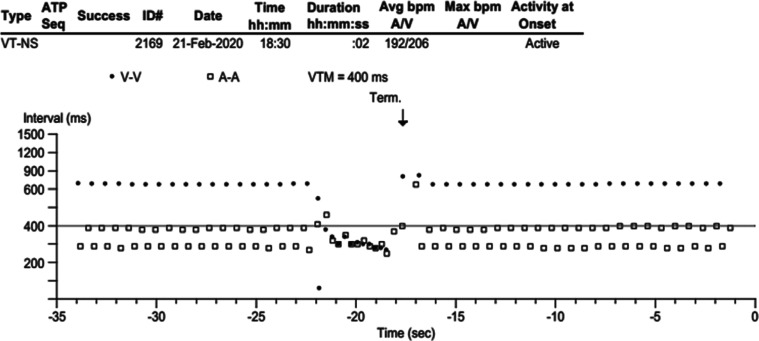
The interval plot of stored electrograms of one of the ventricular high-rate episodes.

A 55-year-old woman with a permanent pacemaker (Advisa DR MRI™, DDDR; Medtronic, Minneapolis, MN, USA) implanted 4 years previously for an infra-Hisian complete heart block (CHB) presented with brief episodes of palpitation lasting for a few seconds to 10 min. She has been 100% dependent on ventricular pacing since the pacemaker implant **(Figure 1)**. The device recorded many atrial high-rate (AHR) and ventricular high-rate (VHR) episodes. One of the VHR episodes was diagnosed as non-sustained ventricular tachycardia (NSVT), the interval plot of which is shown in **Figure 2**. The atrial intervals show an interesting railroad pattern. Given her presentation, we questioned the mechanism of the railroad pattern and how to manage the patient.

## Discussion

As diagnosis options, we considered the following: atrial bigeminy, T-wave oversensing, endless loop tachycardia (ELT), far-field R-wave oversensing (FFRWO), and ventriculo-atrial (VA) conduction. Ultimately, however, the correct answer was FFRWO.

It is important to note that the numbers of A are double the numbers of V during the railroad pattern, and the V–V interval (approximately 700 ms) is equal to the addition of the 2 different atrial intervals (approximately 300 + 400 ms). This can happen if the atrial bigeminy is consistently non-conducted or not tracked. The middle segment of the image in **Figure 2** helps to narrow down the possibilities, which shows that, when the V–V intervals shorten, the railroad appearance is disrupted. In contrast, the intervals return to the railroad pattern coherently with the slowing of V–V intervals. The V–V intervals enter the VHR zone during the period diagnosed as NSVT. It can also be consistent with an atrial run with 1:1 conduction given the identical A–A and V–V intervals. However, in a consistently 100% ventricular pacing (A-paced–V-paced)-dependent patient, it is unlikely, and NSVT with VA conduction is much more likely. It is also possible that the annotated atrial blanking (Ab) during the NSVT episode is an FFRWO rather than a VA conduction.

There is no reason for atrial bigeminy to become altered during NSVT. T-wave oversensing is also extremely rare in atrial leads; instead, it is usually found in intracardiac defibrillator leads. ELT always has a 1:1 atrioventricular (AV) ratio and appears unlike what is seen here. Hence, either an FFFRO or a VA conduction remains the most likely possibility. The variable QRS vector may not be sensed by the atrial lead during VT.

**Figure 3:**
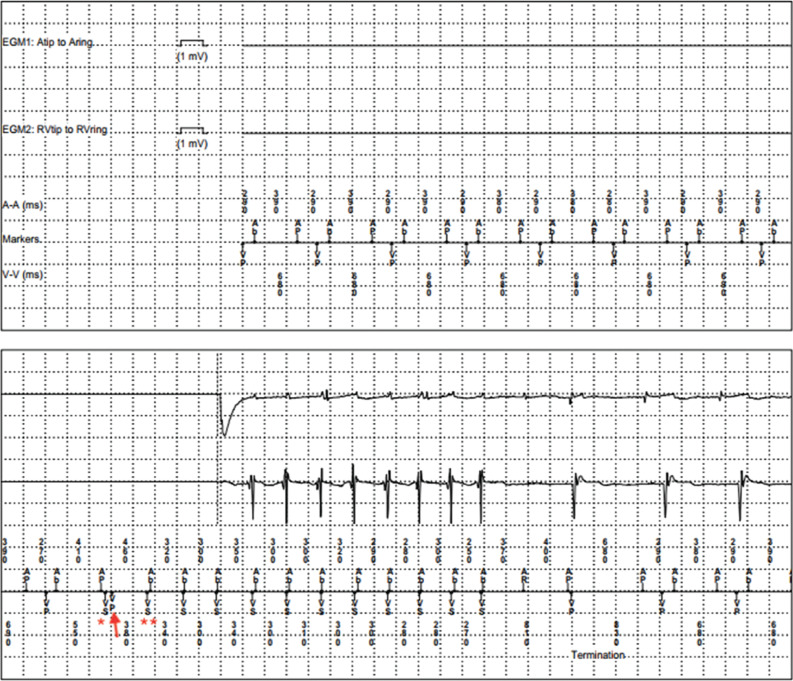
Intracardiac electrograms of the same episode showing non-sustained ventricular tachycardia. The first Vs (*) is followed by another Vp due to “safety pacing” (arrow). The second Vs (**) is immediately followed by Ab. The near-simultaneous Vs–Ab goes against ventriculo-atrial conduction and favors far-field R-wave oversensing. This continues for the next 11 beats. After the termination, the sinus P falls within the post-ventricular atrial refractory period (PVARP) and is binned as AR (in PVARP). The long PVARP (>360 ms) is likely due to a post–premature ventricular complex response.

The stored electrograms (EGMs) offer important insights to analyze the case **(Figure 3)**. The EGMs suggest that the initial rhythm was an A-paced–V-paced rhythm followed by “Ab” (atria in blanking), which can be either a VA conduction or an FFRWO. The differentiation can be difficult at times. Here, FFRWO seems less likely with the VA interval being long (approximately 100 ms). The NSVT helps to pinpoint the actual cause. The first Vs (*) is followed by another Vp due to “safety pacing” (arrow). The second Vs (**) is immediately followed by Ab. The near-simultaneous Vs–Ab goes against VA conduction and favors FFRWO. This continues for the next 11 beats. After the termination, the sinus P falls within the post-ventricular atrial refractory period (PVARP) and is binned as AR (in PVARP). The long PVARP (>360 ms) is likely due to a post-premature ventricular complex (PVC) response.

**Figure 4:**
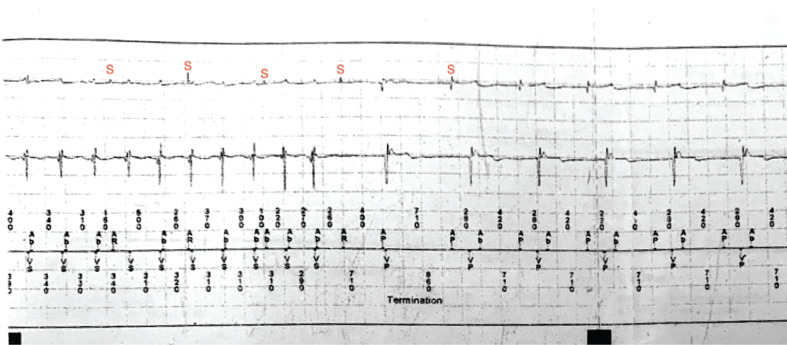
Termination of another non-sustained ventricular tachycardia episode. The S marks the sinus P-waves, which are different in morphology relative to the far-field R-wave oversensing electrograms in the atrial channel.

In this case, all of the AHR episodes were actually related to FFRWO. Termination of another NSVT episode more convincingly proved to be entirely due to FFRWO **(Figure 4)**. The sinus P-waves (marked as “S”) are marching through the FFRWO (different morphology), which again are near-simultaneous as seen in **Figure 3**. The near-simultaneous A and V brings us to the differential of atrioventricular nodal re-entry tachycardia (AVNRT) but is unlikely to have 1:1 AV conduction in the presence of an infra-Hisian CHB. Having said that, we must remember that AVNRT with a retrograde conduction via a fast pathway is still possible, even in the presence of an antegrade CHB. After looking at the EGM in **Figure 3** during railroad with the repetitive sequence of Ap–Vp–Ab, a possibility of atrial non-capture was also considered (analogous to repetitive non–re-entrant ventriculoatrial synchrony). However, the device was checked and proper atrial capture (threshold, 0.75 at 0.4 seconds) was ascertained. There were several episodes of true NSVT, which were medically treated. The patient’s coronary angiogram was normal. A cardiac positron-emission tomography scan is planned to rule out cardiac sarcoidosis in the presence of an AV block and NSVT.

Her P-wave amplitude was consistently 2.4–3 mV (with atrial sensitivity set at 0.3 mV). The sensitivity was reduced (set at 1 mV), which was successful for avoiding the FFRWO. We pondered over the mechanism of the different VA timing of FFRWO during Vp beats versus Vs beats during the NSVT. It was possibly due to the different myocardial breakthrough points (Vp vs. NSVT) leading to dissimilar QRS vectors. This would have led to dissimilar timing of sensing (FFRWO) in the atrial channel. To summarize, these interesting images highlight one of the rarer causes of an atrial railroad appearance.

